# Impact of Molar Distalization with Clear Aligners on Periodontal Ligament Stress and Root Resorption Risk: A Systematic Review of 3D Finite Element Analysis Studies

**DOI:** 10.3390/dj13020065

**Published:** 2025-01-31

**Authors:** Ava Nazeri, Jose A. Castillo, Arash Ghaffari-Rafi

**Affiliations:** 1 School of Dentistry, University of California, San Francisco, CA 94143, USA; 2 Department of Neurological Surgery, School of Medicine, University of California, Davis, Sacramento, CA 95616, USA; jcastillo@ucdavis.edu

**Keywords:** molar distalization, clear aligners, periodontal ligament stress, root resorption, orthodontic biomechanics, three-dimensional finite element analysis, anchorage methods, attachments, class II elastics, mini-implants, stress distribution

## Abstract

**Background/Objectives:** Molar distalization with clear aligners is increasingly used for Class II malocclusions, yet the associated periodontal ligament (PDL) stress and potential root resorption risk remain unclear. Three-dimensional finite element analysis (3D FEA) provides insight into these factors, but variations in attachments and anchorage strategies merit systematic evaluation. To determine whether molar distalization with clear aligners exceeds the PDL stress thresholds for root resorption and to assess how different attachments and anchorage methods influence stress distribution. **Methods:** In accordance with the PRISMA 2020 guidelines, four electronic databases were searched without language or date restrictions. Studies were included if they (1) employed 3D FEA, (2) analyzed PDL stress during aligner-based molar distalization, and (3) assessed root resorption risk or stress thresholds. Two reviewers independently screened and extracted data, yielding eight studies. **Results:** Attachments lowered PDL stress and distributed forces more evenly, reducing root resorption risk compared with no attachment cases. Micro-implants shifted stress to molars and protected anterior teeth; palatal mini-screws achieved greater distalization but higher stress, requiring caution, while buccal mini-screws showed lower stress in first premolar roots. Placing a mini-screw between first and second molars yielded the lowest, most uniform stress. Class II elastics—with precision cuts—demonstrated low compressive stress and improved anchorage, although some resorption risk persisted in maxillary anteriors. **Conclusions:** Clear aligner–based molar distalization can elevate PDL stress to potentially resorptive levels. Although attachments, micro-implants, and Class II elastics improve stress distribution and lessen root resorption risk, it is not fully eliminated. Careful, individualized treatment planning remains essential, and further clinical validation is needed to establish definitive guidelines.

## 1. Introduction

Root resorption is a well-documented yet complex side effect of orthodontic treatment, occurring with variable prevalence and impacting the structural integrity of the root and crown by affecting dentin and cementum [1,2,3]. While typically limited to small, localized areas on the root surface, this process can escalate to irreversible damage under certain conditions. Root resorption is largely attributed to orthodontic forces, which induce mechanical stress and circulatory disturbances within the periodontal ligament (PDL). Despite its clinical significance, the mechanisms driving root resorption remain poorly understood, and its unpredictable nature poses a challenge for clinicians. Factors such as individual susceptibility, genetics, and the mechanical properties of dental tissues further complicate prediction and management.

The severity of orthodontically induced root resorption is influenced by several factors, including the magnitude, direction, and duration of applied force, as well as root morphology and length. Forces in the range of 0.5–1 N (approximately 50–100 gf) are generally considered safe, but forces beyond this range have been associated with increased risk, particularly with intrusive movements, which are more prone to resorption [1,3,4,5,6,7]. Extended appliance time also heightens the likelihood of resorption. However, the optimal force levels remain a topic of debate, with reported values ranging from 0.28 to 3.31 N.

Previous research indicates that clear aligners significantly reduce the risk of root resorption compared to fixed appliances, with a lower prevalence in the aligner group (56.30%) versus the fixed appliance group (82.11%) [8]. Despite their increasing use, questions remain about the biomechanical effects of clear aligners, particularly their impact on periodontal ligament (PDL) stress during molar distalization. High PDL stress may lead to root resorption, underscoring the importance of understanding stress distribution during orthodontic treatment.

The maxillary second molar has reported the lowest accuracy among all teeth and the highest frequency of under-performance, largely because it lacks distal teeth, has a shorter crown, and presents significant challenges for movement with clear aligners. Given these difficulties, studying molar distalization and its effect on PDL stress is crucial in understanding how effectively this tooth can be moved and the biomechanical limitations of clear aligner therapy.

This systematic review addresses whether molar distalization using clear aligners generates PDL stress exceeding the threshold for root resorption and explores how attachment methods and additional anchorage mechanisms—such as Class II elastics, mini-implants, and attachments—affect PDL stress. It aims to evaluate whether these methods minimize stress below the root resorption threshold to optimize treatment outcomes. Zhong et al., 2019 and Moga et al., 2023 [6,9] identified that root resorption is likely when compressive hydrostatic pressure exceeds the capillary blood pressure of 4.7 kPa (0.0047 MPa), reported to range between 0.0047 MPa and 0.016 MPa [6].

To answer these questions, a systematic review of the literature was conducted, focusing on studies using three-dimensional finite element analysis (3D FEA) to assess PDL stress during molar distalization with clear aligners to better understand the biomechanical responses of dental tissues under orthodontic forces. By synthesizing data from relevant studies, this review provides insights to help clinicians enhance patient safety and treatment efficacy.

## 2. Materials and Methods

The systematic review was conducted following the Preferred Reporting Items for Systematic Reviews and Meta-Analyses (PRISMA) guidelines and the Cochrane Handbook for Systematic Reviews of Interventions [4,5,6].

### 2.1. Types of Studies Included

Only studies with human subjects published in English were included, focusing on randomized controlled trials (RCTs), cohort studies, case series, in silico and case–control studies. Reviews, opinion articles, case reports, and case series were excluded. The study’s design was summarized in the PICO format (Population, Intervention, Comparison, Outcome), with the inclusion and exclusion criteria following below.

### 2.2. PICO of the Study

P: Patients requiring molar distalization for orthodontic treatment.

I: Clear aligners.

C: Self-comparison.

O: Stress distribution on the periodontal ligament (PDL), including a quantitative analysis of stress and its clinical implications.

### 2.3. Inclusion Criteria

Studies were selected based on the following criteria:

1. Presence of alveolar bone without disease, degenerative changes, or significant bone loss.
2. Participants with Angle Class II malocclusion.
3. Normal alveolar bone density.
4. No teeth with developmental malformations.
5. Molar distalization as the treatment method.
6. Use of finite element analysis (FEA) and cone-beam computed tomography (CBCT).
7. Measurement of von Mises stress or tensile/compressive stress on the PDL.
8. Systemically and mentally healthy patients with permanent dentition, treated with clear aligner orthodontic therapy.

### 2.4. Exclusion Criteria

The exclusion criteria were as follows:

1. Patients with mixed or deciduous dentition.
2. Individuals who did not receive clear aligner orthodontic treatment.
3. Patients with systemic disorders that increase the risk of infections and negatively affect outcomes.
4. Lack of stress measurement on the PDL.
5. Treatments other than molar distalization.

### 2.5. Outcomes of Interest

The primary outcome of interest was whether the stress on the PDL exceeded the root resorption threshold during molar distalization using clear aligners. Manuscripts were included if they provided stress measurements in the form of von Mises or tensile/compressive stress using 3D FEA. For the simulation of the involved structures on FEA, the physical characteristics were defined as linear elastic, isotropic, and homogeneous for the alveolar bone, periodontal ligament (PDL), and teeth. Secondary outcome variables included the use of attachments, Class II rubber bands, and micro-implants.

### 2.6. Quality Assessment

All included studies underwent quality assessment using a modified version of the Oxford Centre for Evidence-Based Medicine (OCEBM) criteria [10,11]. According to this framework, Level 3 evidence generally corresponds to case–control studies, whereas Level 4 evidence corresponds to case series. Strictly speaking, because each included study relied on a single sample, they could be classified as Level 4 evidence. However, within these studies, each finite element analysis (FEA) simulation effectively introduced distinct comparison groups—often featuring a control group (e.g., no attachments or mini-implants) alongside an intervention group—which aligns more closely with Level 3 evidence. The relative homogeneity of these simulated case studies supports the consistency and reliability of the conclusions drawn, suggesting that this body of evidence aligns with Level 3 according to the OCEBM framework.

### 2.7. Search Strategy

A comprehensive literature search was conducted in Medline (PubMed interface), Embase (Ovid interface), and the Cochrane Central Register for Controlled Trials (CENTRAL; Wiley interface) from the date of database inception to 28 April 2024. Appendix A provides the search protocols, including keywords. Specific search strategies were developed under the guidance of library/information scientists with expertise in systemic review searches. Search results were imported to Covidence (Covidence A/S, Melbourne, Australia) to reduce data entry errors and bias (i.e., deduplicating references). All investigation reports were assessed for inconsistencies (e.g., design description, outcome presentation, and total patients analyzed) [12,13,14,15,16].

## 3. Result

**Figure 1:**
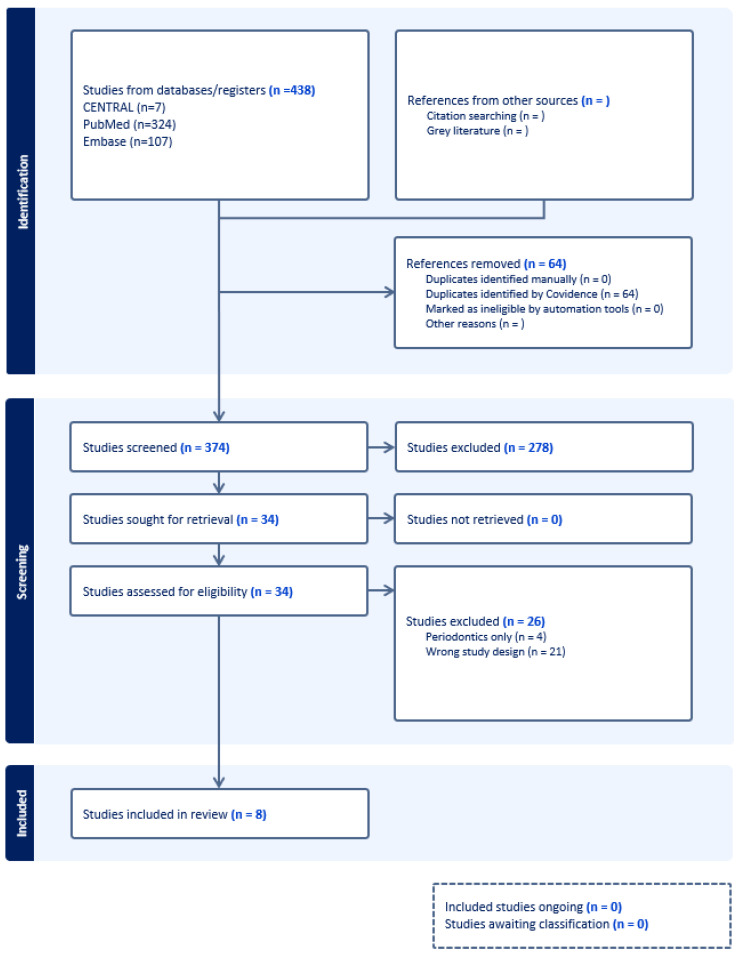
PRISMA flow chart diagram.

A search of Medline, Embase, and CENTRAL identified 438 references, which were screened by title and abstract for eligibility. After excluding 374 studies, 34 articles were assessed in full, resulting in 8 manuscripts that met the inclusion and exclusion criteria. These eight manuscripts focused on patients with Class II malocclusion undergoing molar distalization using clear aligners (Table 1). The identification of studies through databases and registers is detailed in the PRISMA table (Figure 1). 

### 3.1. Study Designs and Outcomes

The characteristics of the study details are summarized in Table 1. Due to the complexity of the tissues involved, cone-beam computed tomography (CBCT) combined with three-dimensional finite element analysis (3D FEA) provided better anatomical accuracy. This simulation process allows for the reconstruction of tissue and precise tooth anatomy, enabling the prediction of stress measurements based on the devices attached to the tooth and the applied load. Data from the eight studies included nine patients.

The studies were categorized based on the devices used in combination with clear aligners to achieve molar distalization. Of the eight studies that evaluated periodontal ligament (PDL) stress, two used attachments, two used Class II elastics with buttons or precision cuts, two used mini-implants, and two employed a combination of attachments and mini-implants. The outcomes of the studies are summarized in Table 2.

### 3.2. Molar Distalization Using Clear Aligners and Attachments

Overall, groups without attachments exhibited the highest levels of tensile, compressive, and Von Mises stress. According to Ayidaga et al., 2021 [17] guideline attachments provided the most controlled stress distribution. In the no-attachment group, the apex of the three roots of the first maxillary molar demonstrated the highest tensile stress (6.5 MPa) and compressive stress (−0.43 MPa).

In the study by Gao et al., 2023 [18], the evaluation of anterior dentition during molar distalization revealed that von Mises stress was higher when two molars were distalized simultaneously compared to a single molar, assuming the same step size. However, reducing the step size for double molar distalization to 0.130 mm resulted in the same reaction force on the anterior teeth as moving one molar at 0.250 mm. The use of horizontal and vertical attachments had a minimal impact on maxillary molar distalization. In the group without attachments, the highest recorded stress was 0.627 MPa, compared to 0.469 MPa with horizontal attachments and 0.468 MPa with vertical attachments. Overall, stress was greater in the group without attachments than in both the horizontal and vertical attachment groups. Therefore, performing double molar distalization with a smaller step size can achieve the same effect as single molar distalization while exerting less stress on the PDL.

### 3.3. Molar Distalization Utilizing Clear Aligner, Class II Elastics, and Button/Precision Cut

The study by Liu et al., 2022 [21] involved models with class II elastics with button or precision cut. Although the Class II elastics decreased stress in the PDL and provided better anchorage control, they did not completely eliminate the risk of PDL stress-induced root resorption, particularly in the maxillary anterior teeth. In all models, the stress exceeded the threshold (−0.0047 MPa), indicating a significant risk of external root resorption.

Among the models, the model where class II elastics were attached to the aligner using precision cutting showed the lowest compressive stress and a more even distribution of stress. This suggests that this method reduces the risk of root resorption in comparison to other methods.

The study by Liu et al., 2023 [22] involved two group sets for sequential molar distalization. In Set I, the second molars were distalized, followed by Set II, where the first molars were distalized after the second molars had moved 2 mm. The models included class II elastics applied either via buttons or precision cuts. The largest compressive stress in the maxillary dentition was observed in Set I at the distal cervical area and distal buccal root apex of the second molar, while the largest tensile stress was found in the mesial cervical area of Set I. In Set II, the highest compressive and tensile stresses occurred on the maxillary first molars, with stress also concentrated in the same region as in Set I, although at a lower value.

### 3.4. Molar Distalization Utilizing Clear Aligners and Micro-Implant/Mini-Screw

The study by Liu et al., 2023 [19] compared the following different anchorage reinforcement models: (1) micro-implant between the second premolar and first molar, (2) button on the canine, and (3) precision cutting on the canine, with 0.25 mm distalization of the second molar in Set I and 0.25 mm distalization of the first molar in Set II. The control models without any anchorage reinforcement showed the highest compressive periodontal ligament (PDL) stress at the anterior region and the lowest at the molars, while models with micro-implants and precision cuts exhibited the lowest anterior stress and the highest stress at the molars. Without anchorage reinforcement, increased compressive stress was observed in the anterior teeth, which may heighten the risk of root resorption and alveolar bone defects. The highest compressive stress across the dentition occurred at the distal cervical surface of the distalizing molars, with notable stress at the labial cervical region and apex of the upper incisors in the anterior region, as well as at the mesiobuccal cervical and apex areas of the canines. Von Mises stress in the alveolar bone was highest at the labial alveolar crest in the anterior region and around the distalizing molars.

The study by Guo et al., 2024 [20] compared the following four groups: (1) control group without mini-screw, (2) direct buccal mini-screw group, (3) direct palatal mini-screw group, and (4) indirect buccal mini-screw group. Among these, the direct palatal mini-screw group exhibited the highest levels of tensile and compressive stress compared to the other groups. All mini-screw groups showed increased molar distalization compared to the control group, with the direct palatal mini-screw group achieving the greatest distal displacement of the maxillary molars. Additionally, the mini-screw groups demonstrated higher levels of maximum compressive stress in the first and second molars than the control group. Specifically, the direct buccal mini-screw group had the lowest tensile and compressive stress in the first premolar roots (0.0928 MPa and 0.0750 MPa), while the indirect buccal mini-screw group showed the highest stress levels in the first premolar roots (0.2921 MPa and 0.3884 MPa). Among all mini-screw groups, the direct palatal mini-screw group recorded the highest tensile and compressive stress in the first and second molars (first molar: 0.2200 MPa and 0.2716 MPa; second molar: 0.4677 MPa and 0.4297 MPa), while also showing the lowest stress levels in the second premolar (0.0688 MPa and 0.0531 MPa).

### 3.5. Molar Distalization Utilizing Clear Aligners and Combinations of Attachment, Micro-Implant, and Button/Precision Cut

The study by Jia et al., 2023 [25] compared the following four groups: (1) control, (2) lingual button, (3) precision cuts, and (4) patient-specific attachment. Across these four models, the distribution pattern of hydrostatic pressure in the PDL was similar, with compressive and tensile stress primarily concentrated on the cervical third and apical third of the root surface. The maximum compressive and tensile stress was found in the maxillary first molar, followed by the second premolar. Stress values progressively decreased from the control group to the patient-specific attachment group. Notably, patient-specific attachments progressively reduced stress concentration in the second premolars, while increasing stress concentration in the first premolars.

The study by Cui et al., 2024 [24] compared the following three groups: (1) no mini-implant, (2) mini-implant placed interradicularly between the second premolar and first molar, and (3) mini-implant placed between the roots of the first and second molars. Different elastic force values were applied as follows: 0, 1, 1.5, and 2 N. The highest periodontal ligament (PDL) stress was observed in the control group without mini-implant reinforcement, while the lowest stress occurred in the group with a mini-implant placed between the first and second molars, which also demonstrated a more uniform stress distribution. The group with a mini-implant between the second premolar and first molar had a less uniform stress distribution compared to the former. Stress around the posterior teeth PDL increased with elastic force, while stress decreased in the incisors and canines. The lateral incisors experienced the minimum stress, whereas the central incisors had the maximum stress (0.0311 MPa). The second molar PDL exhibited higher stress compared to the first molar. A comprehensive chart with values is provided in Table 2. Overall, the worst stress distribution was in the group without mini-implant reinforcement, whereas the most uniform distribution was observed in the group with a mini-implant between the first and second molars.

## 4. Discussion

### 4.1. Overview of Results

This systematic review analyzed whether the periodontal ligament (PDL) stress levels exceed the threshold for root resorption during molar distalization using clear aligners. The threshold values, based on the current literature, suggest that root resorption is likely to occur if stress levels exceed the maximum tolerable stress (MTS) and maximum hydrostatic pressure (MHP). Zhong et al., 2019 and Moga et al., 2023 [6,9] identified that root resorption is likely when compressive hydrostatic pressure exceeds the capillary blood pressure of 4.7 kPa (0.0047 MPa), reported to range between 0.0047 MPa and 0.016 MPa. The reviewed studies used three-dimensional finite element analysis (3D FEA) to measure stress in the PDL, including von Mises and tensile/compressive stresses. The findings consistently indicate that the stress induced during molar distalization often surpasses these critical thresholds, raising concerns about potential risks for external root resorption.

### 4.2. Analysis of PDL Stress in the Context of Molar Distalization

The prevalence of root resorption was significantly lower in the clear aligner group (56.30%) compared to the fixed appliance group (82.11%) as reported by Li et al., 2020, [26]. This finding suggests clear aligners are a better option for reducing the risk of root resorption. However, even though the data suggests a lower prevalence of root resorption with clear aligners, during molar distalization, we still observe stress levels above critical thresholds, indicating the high susceptibility of the PDL to damage in such treatment modalities. The use of attachments, class II elastics, or micro-implants for anchorage reinforcement could potentially aid in stress distribution, but the stress remains above the threshold for root resorption risk.

### 4.3. Effect of Attachments on PDL

The reviewed studies underscore the role of attachments in managing stress distribution within the PDL during molar distalization. Ayidaga et al., 2021 [17] demonstrated that groups without attachments exhibited the highest levels of tensile and compressive stress, with the apex of the maxillary molar roots showing values of 6.5 MPa and −0.43 MPa, respectively, indicating an increased risk of root resorption without attachments. The presence of attachments contributed to a more even distribution of stress, thereby reducing the risks associated with excessive forces.

Gao et al., 2023 [18] showed that distalizing two molars simultaneously led to higher von Mises stress compared to a single molar when using the same step size. However, reducing the step size for double molar distalization to 0.130 mm produced similar stress levels to single molar movement at 0.250 mm, suggesting that smaller step sizes can effectively reduce PDL stress. Stress levels were consistently higher in groups without attachments (0.627 MPa) compared to those with horizontal (0.469 MPa) or vertical attachments (0.468 MPa).

Despite these interventions, it is important to note that the recorded stress levels across all groups were above the threshold for root resorption. Zhong et al., 2019 [9] identified that root resorption is likely to occur when the compressive hydrostatic pressure exceeds the capillary blood pressure of 4.7 kPa (0.0047 MPa), which is reported to be between 0.0047 MPa and 0.016 MPa (Moga et al., 2023 [6]). This emphasizes the need for careful consideration of attachment use and force application during molar distalization to minimize the risk of PDL damage and root resorption.

### 4.4. Influence of Class II Elastics and Buttons on PDL Stress

Liu et al., 2022 [21] demonstrated that Class II elastics provided improved anchorage control; however, they were insufficient in mitigating the risk of stress-induced root resorption, particularly in the maxillary anterior teeth. The study reported compressive stress levels exceeding the root resorption threshold of −0.0047 MPa, as established by Zhong et al., 2019 [9]. This indicates that, despite their utility in orthodontic treatment, Class II elastics can still contribute to stress levels that risk damaging the PDL, especially in areas prone to higher compressive forces. This finding underscores the importance of clinicians carefully monitoring the application of Class II elastics, particularly in patients with a predisposition for root resorption.

Furthermore, Liu et al., 2023 [22] compared the stress profiles between distalizing the second molars versus the first molars, concluding that the distalization of the second molars induced significantly more stress on the PDL. This is a critical finding, as it suggests that targeting different molars can lead to different biomechanical impacts, potentially influencing treatment outcomes and risk profiles. Additionally, no significant difference in stress distribution was observed between using elastics with buttons versus precision cuts, suggesting that the specific attachment mechanism when combined with elastics may be less critical in determining PDL stress than previously thought.

The analysis by Moga et al., 2023 [6] further supported that configurations without Class II elastics resulted in the highest PDL stress, with values exceeding the threshold range from 0.0047 MPa to 0.016 MPa. These results indicate that while Class II elastics do not completely prevent excessive stress, their use reduces the overall PDL stress compared to configurations without them. This reinforces the need for careful planning when designing orthodontic force systems, as poorly controlled forces can lead to detrimental side effects, such as root resorption and damage to the periodontal ligament.

### 4.5. Influence of Micro-Implants on PDL Stress

Liu et al., 2023 [19] found that models without anchorage reinforcement had high compressive PDL stress in anterior teeth, increasing the risk of root resorption and bone defects. Using micro-implants or precision cuts shifted stress to the molars, enhancing distalization efficiency and protecting anterior teeth.

Guo et al., 2024 [20] observed that the direct palatal mini-screw group achieved the greatest molar distalization but also exhibited the highest stress levels, which requires careful management. The direct buccal mini-screw group had lower stress in first premolar roots, suggesting a safer profile for adjacent teeth.

Jia et al., 2023 [23] reported that patient-specific attachments reduced stress on second premolars while increasing it on first premolars, allowing for controlled tooth movement with lower overall stress levels compared to traditional methods.

Cui et al., 2024 [24] demonstrated that placing a mini-screw between the first and second molars resulted in the lowest and most uniform PDL stress. Increasing elastic force amplified stress around posterior teeth but reduced it in incisors and canines, indicating that force adjustments can optimize treatment outcomes.

### 4.6. Limitations

A major limitation of this study is the reliance on three-dimensional finite element analysis (3D FEA). Due to the complexity of tissues and the challenges of in vivo measurement, accurate anatomical reconstruction is simulated using FEA software ABAQUS (6.13-1; Dassault Systèmes Simulia Corp., Stationsplein 8-K, 6221 BT Maastricht, The Netherlands). (version 6.14.1 Dassault Systèmes, Waltham, MA, USA) (version 6.14.1; Dassault Systemes, Velizy-Villacoublay, France), (version 6.11; Dassault Systemes, Velizy-Villacoublay, France). Although this method provides comprehensive biomechanical insights, it cannot fully replicate real clinical scenarios, particularly as it does not account for dynamic masticatory forces. Moreover, FEA is often conducted on a single sample patient rather than across diverse patient populations, further limiting the generalizability of its findings. To enhance the clinical relevance of these results, in vivo studies are needed to validate these findings and address these shortcomings.

This study heavily relies on FEA techniques to evaluate root resorption, employing failure criteria tailored for different material types. For instance, maximum principal stress (S1) and minimum principal stress (S3) [27,28,29,30,31] are frequently used for brittle materials, while von Mises and Tresca stress are better suited for ductile materials [30,31,32], as outlined by yielding theory. These approaches differ in their explanation of material deformation or failure under stress, with maximum hydrostatic pressure typically applied for gasses and liquids. The accuracy of FEA analyses depends on adherence to mandatory boundary conditions and the application of appropriate failure criteria. However, a significant limitation lies in the misalignment between FEA outcomes and the internal microstructure of tissues or their biomechanical responses to forces. This discrepancy often results in findings that deviate from clinical observations, thereby reducing their reliability. While the ductile-like properties of dental tissues, particularly the periodontal ligament (PDL), are well acknowledged, they are not always adequately integrated into such analyses, further contributing to the gap between theoretical models and clinical realities.

### 4.7. Conclusion and Clinical Implication

This systematic review highlights the biomechanical effects of molar distalization using clear aligners, specifically focusing on the impact on the PDL stress. The findings suggest that while clear aligners are generally associated with a lower risk of root resorption compared to fixed appliances, molar distalization often induces stress levels in the PDL that exceed the threshold for root resorption. This underscores the need for careful treatment planning when using clear aligners for molar distalization.

The use of attachments, Class II elastics, and mini-implants has demonstrated potential in redistributing PDL stress more favorably, although they do not completely mitigate the risk of stress-induced root resorption. Notably, attachments contribute to a more even stress distribution, micro-implants improve anchorage, and class II elastics help control stress on the PDL. However, these interventions must be carefully selected and customized for each patient to minimize adverse effects and optimize treatment outcomes.

Ultimately, the choice of attachment type, anchorage reinforcement, and treatment approach should be tailored based on individual patient factors such as bone density, tooth morphology, and periodontal health. Further clinical studies are needed to validate these findings and determine the best strategies for minimizing PDL stress during molar distalization with clear aligners, ensuring safer and more effective orthodontic treatment.

## Figures and Tables

**Table 1:** All clear aligner studies and interventions across included studies, organized by group label, or number (attachment, micro-implant, Class II elastics with button/precision cuts, and combination therapy). The table summarizes the aligner protocols, methodology, classification, and sample size for each intervention. 3D FEA: 3D Finite Element Analysis.

Citation	Intervention	Method	Group-Label/Number Is Based on the Paper	Aligner Details	Method	Classification	Sample Size
Attachment							
Ayidaga et al., 2021 [17]	Single maxillary molar distalization using clear aligner and attachment	Buccal attachment on first Molar, no movement measurement mentioned	No Attachment. Rectangular Vertical Attachment. Guideline Attachment	- Aligner thickness: 0.3 mm - Vertical rectangular attachments: 2.75 mm height, 1.75 mm width, and 1 mm thickness - Guideline attachment: 1.8 mm height, 4 mm width, and 1 mm thickness	3D FEA	class II malocclusion	1
Gao et al., 2023 [18]	Single and double maxillary molar distalization using clear aligner and attachment	Horizontal and vertical attachment on first and second molar. 0.25 mm distalization for single molar distalization, 0.250 mm was set as the control, and the step distance was gradually decreased from 0.250 mm to 0.200 mm, 0.150 mm, 0.140 mm and 0.130 mm, respectively.	1. single molar distalization: horizontal rectangular attachment for 1st and 2nd molar step distance: 0.250 mm. 2. vertical rectangular attachments: for the first and second molars, the step distance: 0.250 mm. - two molar distalization: horizontal rectangular attachment for 1st and 2nd molar, step distance: 0.130 mm two molar distalization: vertical rectangular attachment for 1st and 2nd molar step distance: 0.130 mm	aligner dimension: length × width × thickness: 3 × 2 × 1 mm.	3D FEA	class II malocclusion	1
Micro- Implant							
Liu et al., 2023 [19]	Sequential first and second maxillary molar distalization using clear aligner and micro implant, button, and precision cutting	micro implant between second premolar and first molar, button on canine, precision cutting on canine, 0.25 mm distalization of second molar in set I and 0.25 mm distalization of first molar in set II.	1. No reinforcement group: A1 and A2. 2. Micro implant attached to precision cuts group: B1 and B2. 3. Micro implant attached to buttons group: C1 and C2. - Set 1: 0.25 mm movement of the second molar. - Set 2: 0.25 mm movement of the first molar after the second molar is moved.	button diameter surface: 3 mm, height: 1 mm, micro implants: 8 mm in length, 1.5 mm in diameter, horizontal rectangular attachment = (3 × 2 × 1 mm)	3D FEA	Class II malocclusion	1
Guo et al., 2024 [20]	simultaneous double maxillary molar distalization using clear aligner, mini screw	clear aligners in conjunction with three types of mini screw anchorage and attachments. mini screw: buccal bone between the roots of the maxillary second premolar and first molar. Direct palatal mini screw: palatal alveolar bone 8 mm away from the alveolar crest, between the roots of the maxillary first molar and second molar. Indirect buccal mini screw: buccal alveolar bone, 6 mm away from the alveolar crest, between the roots of the maxillary second premolar and first molar	Control group: no mini screw Direct buccal mini screw group Direct palatal mini screwgroup Indirect buccal mini screw group	Clear aligner: 0.75 mm. vertical attachment: 3 mm in height, 2 mm in width, and 1 mm in thickness. The mini screws were 1.4 mm in diameter and 8 mm in length.	3D FEA	Class II division 1 malocclusion	1
Class II elastics and button/precision cutting							
Liu et al., 2022 [21]	Sequential first and second maxillary molar distalization using clear aligner, class II elastics, button, and precision cutting	vertical rectangular attachment on buccal premolars and horizontal rectangular attachment on second molars, CL II elastics, button on canine, and precision cutting on canine. 2 mm distalization of second molar followed by first molar.	1. No Class II elastics 2. Class II elastics attached to the tooth by buttons 3. Class II elastics attached to the aligner by precision cut - Set I: 0.25 mm initial distalization of the second molar - Set II: Initial distalization of the first molar	The buttons: 3 mm, height 1 mm. vertical rectangular attachments: 2 × 3 × 1 mm, rectangular attachment: 3 × 2 × 1 mm	3D FEA	Class II malocclusion	1
Liu et al., 2023 [22]	sequential first and second maxillary molar distalization using clear aligner, class II elastics, button, and precision cutting	CL II elastics, button on canine and first molar, and precision cutting on canine, 0.25 mm of initial step distance for molar distalization. 2 mm distalization of second molar followed by first molar	1. Class II elastics with button (A1, A2) 2. Class II elastics with precision cut (B1, B2) - Set I: second molar distalization - Set II: first molar distalization after 2 mm second molar distalization	buttons: diameter 3 mm; height 1 mm.	3D FEA	Class II malocclusion	1
Combination of micro-implant with class II elastics and button/precision cut							
Jia et al., 2023 [23]	Simultaneous double maxillary molar distalization with clear aligner, precision cut, lingual button, and patient specific attachment, micro implant	micro implant was located in the interradicular space between the first and second molars.	control lingual button precision cuts patient-specific attachment	lateral hook (thickness= 0.5 mm, length: 9.78 mm), micro implants (diameter, 1.4 mm; length, 8 mm), specific attachments (thickness, 0.5 mm; length, 9.78 mm)	3D FEA	not mentioned	1
Cui et al., 2024 [24]	Simultaneous double maxillary molar distalization using clear aligner, micro implant, angel button, and attachment	vertical attachment from canine to second premolar, interradicular micro implants between the roots of the maxillary second premolar and first molar, and maxillary first molar and second molar at a height of 6mm above the cementoenamel junction, angel button on the bilateral lateral incisors and the canines	1. no micro implant 2. micro implant on interradicular of second premolar and first molar 3. micro implant between the root of 1st and 2nd molars. - Different elastic force values were designed: 0, 1, 1.5, 2 N.	aligner thickness: 0.75 mm—Vertical rectangular attachments 3 mm height, 1 mm thickness, 2 mm upper width and 1.5 mm lower width. first and second molar were distalized by 0.2 mm simultaneously. angel button: between the bilateral lateral incisors and the canines- micro implant: The mini-implants were 1.3 × 7 mm in diameter and length, located between the roots of the maxillary second premolar and first molar, and maxillary first molar and second molar at a height of 6 mm above the cementoenamel junction	3D FEA	class II malocclusion	1

**Table 2:** All clear aligner studies focusing on maxillary molar distalization strategies using clear aligners grouped by intervention type (attachments, mini-implants, Class II elastics, and combined therapies). The table details each study’s citation, methods, experimental groups, highest von Mises/tensile/compressive values, teeth or groups with the highest stress, and key results and conclusions.

Citation	Intervention	Method	Group (Label/Number Is Based on the Paper)	Highest Von Mises/Tensile/Compressive Value	Highest Von Mises Teeth	Highest Von Mises Group	Result and Conclusion
Attachment							
Ayidaga et al., 2021 [17]	Single maxillary molar distalization using clear aligner and attachment	Buccal attachment on first molar, no movement measurement mentioned	No Attachment. Rectangular Vertical Attachment. Guideline Attachment	Highest tensile: 6.5 MPa, Highest compressive: −0.43 MPa	first maxillary molar at the apex of the three roots in the nonattachment group	no attachment group	▪ The group without attachments exhibited the highest levels of both tensile and compressive stress compared to the other groups. ▪ The group with guideline attachments demonstrated the most controlled stress distribution. ▪ All group’s tensile/compressive values were above threshold.
Gao et al., 2023 [18]	Single and double maxillary molar distalization using clear aligner and attachment	Horizontal and vertical attachment on first and second molar. 0.25 mm distalization for single molar distalization, 0.250 mm was set as the control, and the step distance was gradually decreased from 0.250 mm to 0.200 mm, 0.150 mm, 0.140 mm and 0.130 mm, respectively.	1. single molar distalization: horizontal rectangular attachment for 1st and 2nd molar - step distance: 0.250 mm. 2. vertical rectangular attachments: for the first and second molars, the step distance: 0.250 mm. 3. two molar distalization: horizontal rectangular attachment for 1st and 2nd molar, step distance: 0.130 mm 4. two molar distalization: vertical rectangular attachment for 1st and 2nd molar step distance: 0.130 mm	No attachment: 0.627 MPa Vertical Attachment: 0.468 MPa Horizontal Attachment: 0.469 MPa	lateral incisor	significantly higher in group without attachment and with both horizontal and vertical attachment	▪ In the group without attachments, stress levels were significantly higher compared to the other groups. ▪ Distalizing two molars resulted in higher von Mises stress compared to one molar distalization, assuming the same step size. ▪ Reducing the step distance for two molars to 0.130 mm can achieve the same reaction force on the anterior teeth as moving one molar at 0.250 mm. ▪ The use of horizontal and vertical attachments had only minor effects on maxillary molar distalization. ▪ All group’s tensile/compressive values were above threshold.
Mini Implant							
Liu et al., 2023 [19]	Sequential first and second maxillary molar distalization using clear aligner and micro implant, button, and precision cut	micro implant between second premolar and first molar, button on canine, precision cutting on canine, 0.25 mm distalization of second molar in set I and 0.25 mm distalization of first molar in set II.	1. No reinforcement group: A1 and A2. 2. Micro implant attached to precision cuts group: B1 and B2. 3. Micro implant attached to buttons group: C1 and C2. Set 1: 0.25 mm movement of the second molar. Set 2: 0.25 mm movement of the first molar after the second molar is moved	▪ A2 anterior region = 0.041, A1 = 0.308 (above root resorption threshold but below max mentioned). ▪ B1 whole dentition = 0.505, B2 = 0.405.	Not mentioned	A2) anterior B2) molar	▪ In the group without anchorage reinforcement, the highest compressive PDL stress was observed at the anterior area and the lowest at the molars. ▪ In the micro implant attached to precision cut group, the lowest stress was observed at the anterior area and the highest stress at the molars. ▪ Without anchorage reinforcement, compressive stress on the anterior teeth increased, heightening the risk of root resorption and alveolar bone defects.
Guo et al., 2024 [20]	simultaneous double maxillary molar distalization using clear aligner, mini screw	clear aligners in conjunction with three types of mini screw anchorage and attachments. mini screw: buccal bone between the roots of the maxillary second premolar and first molar. Direct palatal mini screw: palatal alveolar bone 8 mm away from the alveolar crest, between the roots of the maxillary first molar and second molar. Indirect buccal mini screw: buccal alveolar bone, 6 mm away from the alveolar crest, between the roots of the maxillary second premolar and first molar	Control group: no mini screw. Direct buccal mini screw group Direct palatal mini screwgroup. Indirect buccal mini screw group.	Direct Palatal Mini Screw: 0.4677 MPa (tensile)	2nd molar teeth	group C: direct palatal mini screw	▪ Compared to the no mini screw group, all groups with mini screws (direct buccal, direct palatal, and indirect buccal) exhibited increased molar distalization, with the direct palatal group showing the greatest distal displacement of maxillary molars. ▪ Mini screw groups showed higher maximum compressive stress in the first and second molars compared to the no mini screw group. ▪ Direct buccal mini screw group: Lowest tensile and compressive stress in the first premolar roots (0.0928 MPa and 0.0750 MPa). ▪ Indirect buccal mini screw group: Highest stress in the first premolar roots (0.2921 MPa and 0.3884 MPa). ▪ Direct palatal mini screw group: Highest tensile and compressive stress in the first and second molars (first molar: 0.2200 MPa and 0.2716 MPa; second molar: 0.4677 MPa and 0.4297 MPa), and lowest tensile and compressive stress in the second premolar (0.0688 MPa and 0.0531 MPa). ▪ Overall, the direct palatal mini screw group had the highest stress levels. ▪ All group’s tensile/compressive values were above threshold.
Class II elastics and button/precision cutting							
Liu et al., 2022 [21]	Sequential first and second maxillary molar distalization using clear aligner, class II elastics, button, and precision cutting	vertical rectangular attachment on buccal premolars and horizontal rectangular attachment on second molars, CL II elastics, button on canine, and precision cutting on canine. 2 mm distalization of second molar followed by first molar.	1. No Class II elastics. 2. Class II elastics attached to the tooth by buttons. 3. Class II elastics attached to the aligner by precision cut. ▪ Set I: 0.25 mm initial distalization of the second molar. ▪ Set II: Initial distalization of the first molar.	All above root resorption threshold	Upper incisors: labial cervical and apex Canine: mesio-buccal cervical and apex	No class II elastics	▪ Class II elastics attached to the aligner using precision cutting showed the lowest compressive stress and a more even distribution of stress, suggesting a reduced risk of root resorption compared to other methods.
Liu et al., 2023 [22]	sequential first and second maxillary molar distalization using clear aligner, class II elastics, button, and precision cutting	CL II elastics, button on canine and first molar, and precision cutting on canine, 0.25 mm of initial step distance for molar distalization. 2 mm distalization of second molar followed by first molar	1. Class II elastics with button (A1, A2) 2. Class II elastics with precision cut (B1, B2) ▪ Set I: second molar distalization ▪ Set II: first molar distalization after 2 mm second molar distalization		Maxillary second molar	Set I: second molar distalization using CL II elastics with either button or precision cutting	▪ The highest PDL stress occurred during second molar distalization in Set I using Class II elastics with either button or precision cutting. ▪ The second largest stress was on the maxillary first molars in Set II, with lower stress on the maxillary second molars in Set I.
Combination of micro-implant, class II elastics, and button/precision cut							
Jia et al., 2023 [23]	Simultaneous double maxillary molar distalization with clear aligner, precision cut, lingual button, and patient specific attachment, micro implant	micro implant in the interradicular space between the first and second molars.	control lingual button precision cuts patient-specific attachment	no value found	maxillary first molar followed by 2nd premolar	Control group	▪ For all four models, hydrostatic pressure distribution in the PDL was similar, with compressive/tensile stress concentrated on the cervical and apical thirds of the root. ▪ Maximum stress was observed on the maxillary first molar, followed by the second premolar. ▪ Stress value progressively decreased from control to patient-specific attachment groups. ▪ Patient-specific attachments reduced stress on the second premolars but increased it on the first premolars.
Cui et al., 2024 [24]	Simultaneous double maxillary molar distalization using clear aligner, micro implant, angel button, and attachment	vertical attachment from canine to second premolar, interradicular mini-implants between the roots of the maxillary second premolar and first molar, and maxillary first molar and second molar at a height of 6mm above the cementoenamel junction, angel button on the bilateral lateral incisors and the canines	1. no micro implant 2. micro implant on interradicular of second premolar and first molar 3. micro implant between the root of 1st and 2nd molars. ▪ Different elastic force values were designed: 0, 1, 1.5, 2 N.	0.0311 MPa	Central incisor and second molar stress in micro implant	no micro implant	▪ No micro-implant group had the highest stress levels compared to other groups. ▪ micro-implant group (between first and second molar) showed well-distributed stress.

## Data Availability

The raw data supporting the conclusions of this article will be made available by the authors, without undue reservation.

## Abbreviation

PDL	Periodontal Ligament
FEA	Finite Element Analysis
3D	Three-Dimensional
CBCT	Cone-Beam Computed Tomography
PRISMA	Preferred Reporting Items for Systematic Reviews and Meta-Analyses
LoE	Level of Evidence
MTS	Maximum Tolerable Stress
MHP	Maximum Hydrostatic Pressure
RCT	Randomized Controlled Trials
OSA	Obstructive Sleep Apnea
MPa	Megapascal

## Appendix A

Pubmed (MEDLINE) Search Strategy

(((aligner OR aligners) OR (brace OR braces)) OR (Invisalign)) AND (((periodontal) AND (membrane OR membranes)) OR (((alveolodental) OR (periodontal) OR (dental)) AND (ligament OR ligaments)))

Embase Ovid Search Strategy

(1) aligner OR aligners (2) brace OR braces (3) invisalign (4) #3 OR #2 OR #1

(5) alveolodental OR periodontal OR dental (6) ligament OR ligaments

(7) #5 AND #6 (8) membrane OR membranes (9) periodontal (10) #8 AND #9 (11) #7 OR #10

(12) #11 AND #4

CENTRAL Search Strategy

(1) aligner * (2) brace * (3) invisalign (4) #3 OR #2 OR #1

(5) alveolodental OR periodontal OR dental (6) ligament * (7) #5 AND #6 (8) membrane *

(9) periodontal (10) #8 AND #9 (11) #7 OR #10 (12) #11 AND #4
